# The Fleming Memorial Hospital for Sick Children, Newcastle-On-Tyne

**Published:** 1892-09-24

**Authors:** 


### THE FLEMING MEMORIAL HOSPITAL FOR SICK CHILDREN, NEWCASTLE-ON-TYNE.

[By Our Special Commissioner.]

We have no doubt that those who are immediately responsible for the administration of this hospital are as desirous as anyone well can be to secure every comfort and protection for the little sufferers who seek relief at the institution under their control. This, we presume, may be taken for granted, and we commence our report with the statement because it will be necessary for us to give a picture of the Fleming Memorial Hospital as it actually presents itself to the eye of a visitor who has a technical acquaintance with its condition, as it ought to present itself—a picture which cannot fail to startle, if it does not even shock the managers. It certainly startled and shocked the writer, and it will, we hope, arouse and fix the attention of the public in Newcastle and the neighbourhood.

The hospital was rebuilt upon the present most excellent site so recently as 1888. Not only has money not been stinted, but it has been lavishly expended, as the elaborate furniture and decorations of the board-room, and the large, well-proportioned, but useless dining-room, prove to demonstration. In every part of the hospital money, we repeat, has been freely expended. Floors, fittings, furniture, all show this to be so, but it is understood, and it will not, we believe, be contradicted, that those who were responsible for the planning and construction of the new buildings possessed little or no technical knowledge, and thus a heart-breaking failure is the result. Even the kitchens testify to this fact eloquently. Only a small portion of the extensive ranges and other fittings are found necessary, the others being unused, and they will gradually, no doubt, fall into disrepair and become anything but attractive objects.

We were informed that on several occasions steps have been taken to re-adjust the drainage which has from the first been unsatisfactory. There was patent evidence of the unhealthiness of the institution at the time of our visit, in the languid aspect of the children, supported by the presence of erysipelas in the wards. There was additional evidence which came under our notice, which we refrain from quoting here. The wards, though they contain, as we have said, handsome furniture, *i.e.*, brass cots and expensive fittings, are too narrow for sick rooms, and sadly overcrowded, that is to say, there is not an adequate superficial area per cot. We are sorry to have to say all this, but we think it is only right to speak out in the interests of the poor little sufferers, and also of the Committee, who can have no knowledge of the real dangers which the children under their charge are unnecessarily undergoing within the walls of this institution.

The Fleming Memorial Hospital, with its lavish expenditure, its modern buildings, and everything which, on paper, would seem to make for efficiency, presents a forcible illustration of the truth that money cannot of itself produce excellence, effect recoveries, or secure the proper treatment of hospital patients. Under the actual conditions to be found in the two institutions, it is far better to be a patient in the Royal Infirmary, which is admittedly out of date, many of its buildings being ill adapted to modern requirements, than to be placed in a ward of the Fleming Memorial Hospital, though it was rebuilt only three years ago. Patients do not need gilded chambers, especially if death dangers are unpleasantly adjacent, but they do require intelligence in administration, adequate sanitary and hygienic surroundings, and the enforcement of a system which will promote recovery as rapidly as possible. Hence the Fleming Memorial Hospital as it presents itself to the expert to-day is an institution to weep and lament over seeing that the startling faults to be found within its walls are all capable of easy remedy, and would have been entirely prevented if the necessary knowledge had been forthcoming when the plans were first settled, provided a trained administrative had been available from the moment the hospital commenced to admit patients for treatment.

We believe, as public trustees, that the Committee would be well advised to close the Fleming Memorial Hospital altogether for a while in order to have the whole of the drainage carefully examined and reconstructed if necessary; to take steps to fumigate and disinfect the whole of the infirmary and buildings, and to pass a resolution to reduce the number of the cots in the larger wards by taking out three, or, better still, four of those at present to be found there. It is, of course, a pity the wards are not wider. Being as they are, it is necessary to reduce the number of cots so as to secure an adequate superficial area and cubic space per bed. This is the only possible step, and one which should be taken without a moment's delay.

As it is, a building which might have been under happier circumstances a just pride to everyone connected with it, has become little better than a dangerous receptacle for sick children, who certainly deserve, and will, no doubt, now that attention has been called to the circumstances, promptly secure a better fate.

